# A Structure-Activity Relationship Comparison of Imidazodiazepines Binding at Kappa, Mu, and Delta Opioid Receptors and the GABA_A_ Receptor

**DOI:** 10.3390/molecules25173864

**Published:** 2020-08-25

**Authors:** Guanguan Li, Amanda N. Nieman, Md Yeunus Mian, Nicolas M. Zahn, Brandon N. Mikulsky, Michael M. Poe, Kashi R. Methuku, Yongfeng Liu, James M. Cook, Douglas C. Stafford, Leggy A. Arnold

**Affiliations:** 1 Shenzhen Key Laboratory of Small Molecule Drug Discovery and Synthesis, Department of Chemistry, College of Science, Southern University of Science and Technology, Shenzhen 518055, China; guanguan.li@yahoo.com; 2 Department of Chemistry and Biochemistry and the Milwaukee Institute for Drug Discovery, University of Wisconsin-Milwaukee, Milwaukee, WI 53201, USA; amanda.nieman15@gmail.com (A.N.N.); mmian@uwm.edu (M.Y.M.); nmzahn@uwm.edu (N.M.Z.); kashireddy.m@gmail.com (K.R.M.); capncook@uwm.edu (J.M.C.); dcstaff@uwm.edu (D.C.S.); 3 Pantherics Incorporated, La Jolla, CA 92037, USA; mikulsky@pantherics.com; 4 Department of Chemistry, Western Michigan University, Kalamazoo, MI 49008, USA; michael.poe@wmich.edu; 5 National Institute of Mental Health Psychoactive Drug Screening Program, Department of Pharmacology, University of North Carolina Chapel Hill, Chapel Hill, NC 27599, USA; yongfeng@email.unc.edu

**Keywords:** opioid receptor, imidazodiazepine, GABA_A_ receptor

## Abstract

Analgesic and anti-inflammatory properties mediated by the κ opioid receptor (KOR) have been reported for oxadiazole imidazodiazepines. Affinities determined by radioligand competition assays of more than seventy imidazodiazepines using cell homogenates from HEK293 cells that overexpress KOR, µ opioid receptor (MOR), and δ opioid receptor (DOR) are presented. Affinities to synaptic, benzodiazepine-sensitive receptors (BZR) were determined with rat brain extract. The highest affinity for KOR was recorded for GL-I-30 (Ki of 27 nM) and G-protein recruitment was observed with an EC_50_ of 32 nM. Affinities for MOR and DOR were weak for all compounds. Ester and amide imidazodiazepines were among the most active KOR ligands but also competed with ^3^H-flunitrazepam for brain extract binding, which is mediated predominately by gamma aminobutyric acid type A receptors (GABA_A_R) of the α_1-3_β_2-3_γ_1-2_ subtypes. Imidazodiazepines with carboxylic acid and primary amide groups did not bind KOR but interacted strongly with GABA_A_Rs. Pyridine substitution reduced KOR affinity. Oxadiazole imidazodiazepines exhibited good KOR binding and interacted weakly with BZR, whereas oxazole imidazodiazepines were more selective towards BZR. Compounds that lack the imidazole moiety, the pendent phenyl, or pyridine substitutions exhibited insignificant KOR affinities. It can be concluded that a subset of imidazodiazepines represents novel KOR ligands with high selectivity among opioid receptors.

## 1. Introduction

The κ opioid receptor (KOR) belongs to a class of opioid receptors that include the µ and δ opioid receptor (MOR and DOR) and nociception opioid receptor (NOP) [1]. Drug candidates targeting this G protein-coupled receptor (GPCR) have been developed for neuropsychiatric disorders [2] and pain [3,4]. The concept of biased agonism [5] that distinguishes between GPCR activation and β-arrestin mediated signaling pathways has shown promise in the development of KOR-based analgesics with fewer side effects [6,7]. This is important for the development of new treatments for neuropathic pain (NP), which occurs in about 7% of the US population [8]. NP can arise without overt stimulation of peripheral sensory neurons and is usually associated with other diseases such as diabetic neuropathy, infections, and cancer chemotherapies [9].

We introduced imidazodiazepine GL-IV-03 as a new drug candidate for NP, which alleviated the agitation response in both phases of the formalin nociception test without inducing impairment of sensorimotor coordination [10]. GL-IV-03 interacted selectively with KOR and reduced the production of nitric oxide (NO) by activated microglia. The anti-inflammatory response was reversed in the presence of KOR antagonist norbinaltorphimine. Microglia have been implicated in the maintenance of NP by transiting from resting (ramified) to an activated, amoeboid morphology [11] followed by the secretion of pro-inflammatory molecules including NO [12,13]. Microglia affected dorsal horn astrocytes and acted synergistically to incite and maintain NP [11]. The role of NO in pain has been demonstrated by injection of NO [14,15].

In support of our therapeutic pain research, a large library of imidazodiazepines has been screened in collaboration with the National Institute of Mental Health’s Psychoactive Drug Screening Program to identify high-affinity KOR ligands. Here, we present affinities of these compounds at the KOR, MOR and DOR and the brain benzodiazepine binding site, discuss their structure-activity relationships, and formulate a KOR binding model for imidazodiazepine GL-I-30.

## 2. Results

Ligand affinities were determined for KOR, DOR, and MOR using radioligand binding assays at a screening concentration of 10,000 nM [16]. For these assays, cell homogenates from HEK293 cells that overexpressed KOR, MOR, and DOR were used in combination with [^3^H]-U69593, [^3^H]-DAMGO, and [^3^H]-DADLE, respectively. Compounds that exceeded more than 50% radioligand displacement were investigated further in concentration-dependent experiments to determine K_i_ values. Gamma aminobutyric acid type A receptor (GABA_A_R) binding was determined with rat brain homogenate and ^3^H-flunitrazepam. Flunitrazepam binds to GABA_A_R subtypes that consists of two α and two β subunits and one γ or δ subunit [17]. Strong affinities to α_1-3,5,6_β_1-3_γ_1-y_/δ GABA_A_Rs have been reported for flunitrazepam [17,18,19,20]. The extrasynaptic GABA_A_R subtype expression in the brain includes 43% α_1_β_2_γ_2_, 15% α_2_β_3_γ_2_ + 8% α_2_βγ_1_, 10% α_3_β_3_γ_2_, 6% α_4_βγ/δ, 4% α_5_β_3_γ_2_, and 4% α_6_β_2_γ_2_/δ [21]. Therefore, compounds with high affinity toward the synaptic benzodiazepine-sensitive receptors (BZR) predominately bind α_1-3_β_2-3_γ_1-2_ GABA_A_Rs. Alternatively, compounds with weak BZR binding might be selective for α_4-6_βγ/δ GABA_A_Rs. Detailed GABA_A_R subtype binding for several imidazodiazepines, as well as *in vivo* evaluations, can be found in the publications cited.

Some imidazodiazepines that were developed originally as anxiolytic drug candidates with weak affinity to α_1_β_2_γ_2_ and good affinity to α_2,3,5_β_3_γ_2_ GABA_A_Rs exhibited surprisingly strong KOR affinities. Table 1 summarizes carboxylic acid derivatives of chiral and achiral imidazodiazepines bearing a 2’-fluorophenyl ring.

The imidazodiazepine with the highest measured KOR affinity was GL-I-30 (K_i_ = 27 nM) (Table 1, entry 1). Interestingly, it was the only ligand in the series that also exhibited an appreciable affinity for MOR (K_i_ = 1850 nM). Overall, compounds with a (S) methyl configuration were superior ligands for KOR. The affinity difference between (R) and (S) ligands ranged between 1.3 and 4.9-fold (Table 1, entries 5 vs. 9 and 13 vs. 28). Four different R_1_ substituents were explored. For entries 4, 5, and 10, we found that cyclopropyl was superior to bromo and acetylene. Other examples supporting this SAR were entries 20 vs. 28 and 26 vs. 29. For R_2_ (esters, thioesters and amides) we observed that a large hydrophobic group like *t*-butyl was a better fit for KOR’s binding pocket than smaller substituents such as propyl, ethyl, or methyl (Table 1, entries 1, 6, 10, and 13). The KOR affinity difference between *t*-butyl and methyl ester was 4.5-fold. Interestingly, the change from an ethyl to a trifluoroethyl ester reduced KOR affinity by 4.5-fold (Table 1, entries 10 vs. 25). A similar trend was observed for amides. The change from a *t*-butyl to methyl amide reduced KOR binding by 3.1-fold (Table 1, entries 3 vs. 12). The thioester GL-I-77 exhibited KOR affinity similar to the corresponding ester (Table 1, entries 10 vs. 14). *N,N*′-Dimethyl amides were poor KOR ligands. The change from an *N*-methyl amide to *N,N*′-dimethyl amide reduced KOR affinity by 6.6-fold (Table 1, entries 12 vs. 29). Non-substituted amides as well as carboxylic acid ligands exhibited very low KOR affinities. No KOR affinity was observed for GL-II-93 (MIDD0301) (Table 1, entry 37). Interestingly, the BZR affinity for non-substituted amide and carboxylic acid imidazodiazepines was below 100 nM (Table 1, entries 32–37). The R_1_ cyclopropyl group that improved KOR affinity significantly reduced BZR affinity in comparison to a bromo substituent (Table 1, entries 4 vs. 5, 9 vs. 17 and 26 vs. 30). Similar to KOR, (S) methyl imidazodiazepines showed better BZR affinities than (R) isomers. In some cases the affinity difference was 5.6-fold (Table 1, entries 29 vs. 31).

For the same scaffold, the pendent aromatic ring had a significant impact on KOR binding. The binding of ligands with a 2′-pyridine substituent are summarized in Table 2.

Compounds with a 2′-pyridine substituent exhibited a lower KOR affinity than those bearing a 2′-fluorophenyl substituent. The difference ranged between 2-4.7-fold (Table 2, entry 2 vs. Table 1, entry 9). Similar to compounds in Table 1, ligands with larger hydrophobic groups were more potent (e.g., ethyl ester GL-II-19 vs. methyl ester GL-II-32) (Table 2, entry 4 vs. 6). Carboxylic acid ligands showed weak KOR affinity (Table 2, entries 8–10). Interestingly, achiral imidazodiazepines, such as HZ-166 and MP-III-024, exhibited slightly stronger affinities to KOR than their chiral counterparts with (R) configurations (Table 2, entries 3 vs. 4 and 5 vs. 6). For substitutions at R_1_, compounds with a bromo function were more active than those with an acetylene group (Table 2, entries 2 vs. 4 and 8 vs. 10). In contrast, 2′-pyridine bearing ligands exhibited good to excellent BZR binding. Achiral ligands MP-II-68 and SR-II-54 (Table 2, entries 1 and 9) were especially active. Also in this series, carboxylic acid derivatives were good BZR ligands (Table 2, entries 8–10).

Ester and amide bioisosteres were investigated next. The binding for a series of oxadiazoles is summarized in Table 3.

The imidazodiazepine with the strongest KOR affinity in this series was GL-I-81 (Table 3, entry 1). It was the only ligand in this series that exhibited appreciable MOR affinity (Ki = 2920 nM). Interestingly, imidazodiazepine oxadiazoles exhibited SAR similar to imidazodiazepine esters and amides. 2′-Fluorophenyl substituted compounds showed better KOR affinities than corresponding 2′-pyridine ligands (Table 3, entries 9 vs. 10). Isopropyl substituted oxadiazoles were more active than the corresponding ethyl or methyl substituted imidazodiazepine oxadiazoles (Table 3, entries 1, 3 and 4). No significant difference in KOR affinity was found between the methyl and ethyl substitution (Table 3, entries 3 vs. 4 and 11 vs.12). Compounds with a (S) methyl configuration exhibited better KOR affinities than the corresponding (R) ligands (Table 3, entries 1 vs. 2 and 4 vs. 7). For R_1_ substitution, bromo was superior to acetylene and cyclopropyl (Table 3, entries 6, 7, and 9). All oxadiazoles exhibited better affinities towards KOR than BZR, except achiral ligands with a 2′-pyridine substituent (Table 3, entries 11 and 12).

Next oxazole imidazodiazepines were explored. Their binding is summarized in Table 4.

Imidazodiazepine oxazoles with a bromo substituent in the R_1_ position were superior KOR ligands in comparison to those bearing an acetylene group (Table 4, entries 1 vs. 4, 2 vs. 7, 3 vs. 6, 5 vs. 9, 8 vs. 11, 12 vs. 13 and 14 vs. 15). Ligands with a methyl substituted oxazole were less active than those with a non-substituted oxazole (Table 4, entries 1 vs. 5, 8 vs. 9, 4 vs. 10, 12 vs. 14, and 13 vs. 15). Also, similar to all other scaffolds, compounds with a 2′-fluorophenyl group exhibited greater KOR affinities than those with phenyl or 2′-pyridine substitutions (Table 4, entries 1, 8 and 12, and entries 5, 9 and 14). Achiral oxazole ligands exhibited better affinities toward KOR than chiral ligands (Table 4, entries 1 vs. 2 and 4 vs. 6). The configuration of the R_3_ methyl group did not significantly influence KOR binding (Table 4, entries 2 vs. 3 and 6 vs. 7). Achiral oxazoles exhibited better BZR affinities than chiral oxazoles, especially those bearing phenyl or 2′-fluorophenyl substitutions. Chiral imidazodiazepine oxazoles with a (S) configuration were better ligands for BZR than their corresponding (R) isomers (Table 4, entries 2 vs. 3 and 6 vs. 7). Imidazodiazepines with a methyl substituted oxazole exhibited slightly lower BZR affinities than those without. Finally, 2′-pyridine substitution resulted in lower BZR affinities for imidazodiazepine oxazoles.

**Figure 1:**
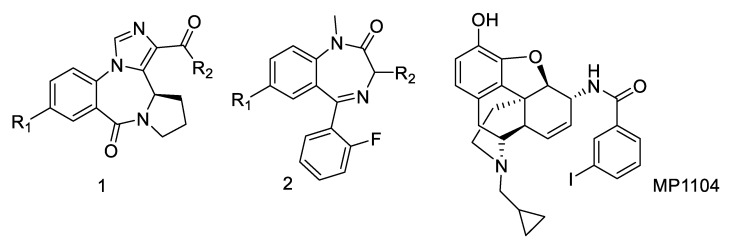
Benzodiazepine compound series and opioid receptor agonist MP1104.

Other related benzodiazepines have been investigated for KOR affinity but did not exhibit significant KOR activity at 10,000 nM (Figure 1, compounds **1** and **2**).

A series of compounds that lack a pendent phenyl ring and exhibit selective α_4_β_3_γ_2_ GABA_A_R binding was described as potential new treatments for asthma (Figure 1, **1**) [22,23,24]. Among a series of more than thirty compounds, none exhibited significant KOR affinity. Benzodiazepines lacking the imidazole moiety such as **2 [25]** did not show any significant KOR affinity either.

To demonstrate that GL-I-30 is a full KOR agonist, a BRET recruitment assay was employed [26].

**Figure 2:**
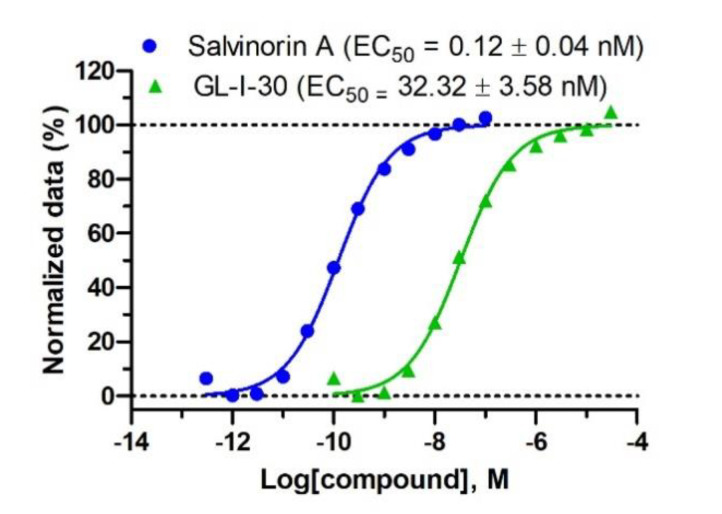
BRET recruitment assay. HEK293 cells were transfected with Gα_oA_-RLuc, Gβ3, Gγ8-GFP2 and human KOR and treated with increasing concentrations of GL-I-30 or full agonist salvinorin A. Luminescence ratio data at 400 nm and 515 nm were normalized to vehicle and 10 nM salvinorin A. Non-linear regression was used to determined EC_50_ values. Data n = 24 is depicted as mean and SEM.

GL-I-30 induced the recruitment of Gα_oA_ protein to KOR with an EC_50_ of 32.3 nM (Figure 2). The efficacy was 100% in comparison to full agonist salvinorin A.

**Figure 3:**
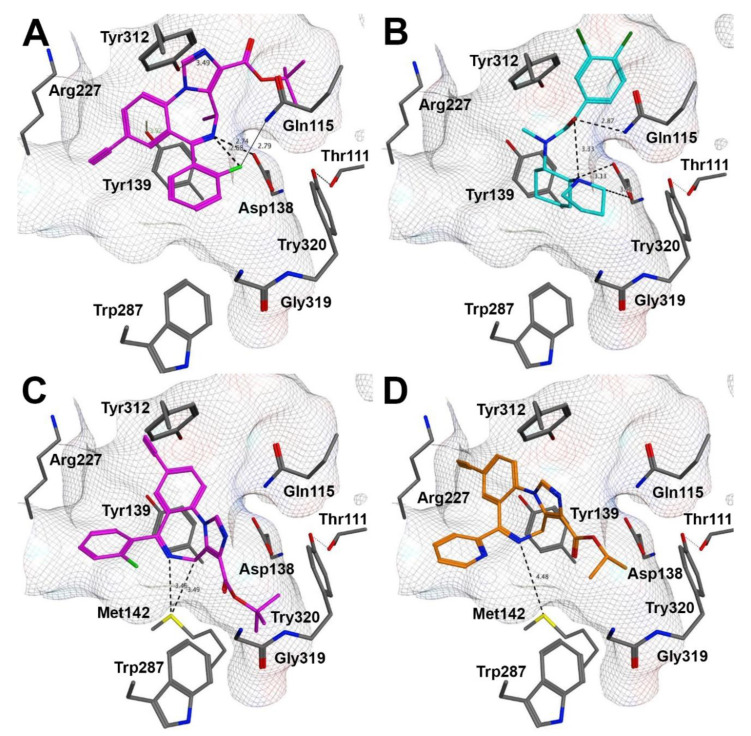
Compound docking poses with KOR [PDB ID 6B73] [27]. (**A**) Best docking pose for GL-I-30, (**B**) recreated docking pose for U50,488, (**C**) second best docking pose for GL-I-30, (**D**) best docking pose for MP-II-068.

To correlate the SAR of imidazodiazepines with available structural information about KOR, we used the recently reported active-state KOR structure [27]. The majority of high affinity KOR ligands have a basic nitrogen that enables hydrogen bonding with Asp138. MP1104 (Figure 1) was used for the crystallization of the active-state KOR structure due to its superior ability to promote the recruitment of nanobody NB39 to KOR. GL-I-30 was docked into the binding pocket resulting in two possible docking poses (Figure 3A,C).

Like MP1104, GL-I-30 was able to form a salt-bridge with Asp138 for the best docking pose (Figure 3A). The calculated imine pK_a_ of GL-I-30 (7.2) is highly dependent on the alpha substituent (chiral methyl) and the pendent phenyl ring. The calculated imine pK_a_ of MP-II-068 bearing a 2′-pyridine substituent is significantly lower than 6.7 and the affinity towards KOR is 4.9% of the GL-I-30- KOR binding affinity (Table 2, entry 1). These electronic effects also resulted in an alternative KOR docking pose for MP-II-068 in comparison to GL-I-30 (Figure 3, A vs. D).

Further stabilization by the 2′-fluorophenyl substituent of GL-I-30 through intramolecular hydrogen bonding can occur [28], as well as interaction with Gln115 (2.79 Å), although intermolecular H…F bonds are very weak (Figure 3A) [29]. Ligand interactions with Gln115 were also observed for U50,488 [30], a highly selective KOR agonist, when docked into the MP1104 binding pocket (Figure 3B) [27]. Many classic opioids interact with Tyr139 of KOR [31]. A possible interaction of Tyr139 with the ether oxygen of MP1104 and salvinorin A was reported [27]. For GL-I-30, we observed π-hydrogen bonding with Tyr139. The iodobenzamide group MP1104 filled out the same pocket as the *t*-butyl ester of docked GL-I-30. Interestingly, MP1104 analogs with different benzamide groups exhibited similar KOR affinities, supporting the fact that this pocket is highly inducible [27]. Also for GL-I-30 analogs with different esters and amides we observed similar KOR affinities. Hydrogen bonding between the benzamide of MP1104 and Tyr312 appeared to be mediated by water (3.6 Å). The distance between the imidazole nitrogen of GL-I-30 and Tyr312 is 3.49 Å, enabling a similar stabilization.

A lower docking score was observed for an alternative orientation of GL-I-30 (Figure 3C). The low affinity KOR ligand MP-II-068 assumed this orientation as the best docking pose (Figure 3D). The ester function of both ligands occupied the hydrophobic pocket shaped by Trp287, which was occupied by the MP1104 cyclopropylmethyl group. This residue is linked to a Pro-Ile-Phe motif, which is a central switch for GPCR activation [32]. In this alternative pose, GL-I-30 can interact with Met142. A similar occupancy was observed for biased agonist IBNtxA, when docked into KOR [27].

## 3. Discussion

Affinity data for a large library of imidazodiazepines contribute to a comprehensive SAR that indicates similar trends of opioid receptor binding for carboxylic acid derivatives and their bioisosteres. The greatest affinities towards KOR were imidazodiazepine esters and amides with large hydrophobic substituents and a 2′-fluorophenyl group. Larger R_1_ groups such as cyclopropyl and bromo increased KOR binding and among chiral ligands the (S) isomer was superior. KOR binding affinities of oxadiazole and oxazole imidazodiazepines were lower than ester and amide imidazodiazepines.

The most active imidazodiazepine, GL-I-30, was confirmed as a full KOR agonist inducing the recruiting of G-protein Gα_oA_ to KOR with an EC_50_ of 32 nM, correlating well with the K_i_ of 27 nM for KOR affinity. The opioid receptor selectivity of GL-I-30 in comparison to MOR is 68-fold. In respect to BZR binding a 6.5-fold selectivity was observed. Thus, the binding pocket of KOR and GABA_A_Rs have a commonality that can be deduced from the presented data. Several ligands have equal affinities for BZR and KOR, however, some of these ligands exhibit excellent GABA_A_R subtype selectivity. For example, SH-053-2′F-S-CH_3_ is a selective α_2,3,5_β_3_γ_2_ GABA_A_R ligand with low efficacy for the α_1_β_3_γ_2_ GABA_A_R [33]. Thus, biochemical studies are warranted to further characterize these compounds. For BZR binding, GABA_A_R subtype selectivity can be determined by electrophysiology to identify selective α_2,3,5_β_3_γ_2_ GABA_A_R ligands with several clinical applications including pain [34,35]. For KOR agonists, G-protein biased ligands with weak effects on β-arrestin recruitment have shown promise as analgesic agents with reduced adverse side effects, such as sedation and dysphoria [36]. GPCR downstream signaling such as GTP hydrolysis, β-arrestin recruitment, and cAMP inhibition have to be evaluated for ligands of interest.

Medicinal chemistry research resulted in many high affinity GABA_A_R ligands with insignificant KOR affinity. Two of them are under development as clinical candidates for epilepsy (KRM-II-81) [37] and asthma (GL-II-93 also known as MIDD0301) [38]. In contrast, only GL-I-76 (Table 1, entry 23) and GL-III-63 (Table 3, entry 9) have low BZR affinities and moderate affinity towards KOR with 371 nM and 678 nM, respectively. Current research efforts are focused on designing more selective imidazodiazepines with better KOR affinities and developing these novel anti-inflammatory agents such as GL-IV-03 for neuropathic pain.

## 4. Materials and Methods

### 4.1. Synthesis 

The synthesis of the majority of ligands in Table 1, Table 2 and Table 4 has been described in Li et al. [39,40] The synthesis of imidazodiazepine oxadiazoles (Table 3) has been described in Cook et al. [41,42].

### 4.2. Radioligand Binding Assays 

Detailed protocols for the primary and secondary radioligand binding assays can be found in the National Institute of Mental Health’s Psychoactive Drug Screening Program (NIMH PDSP) Assay Protocol Book [43]. Briefly, primary and secondary radioligand binding assays are carried out in a final of volume of 125 μL per well in appropriate binding buffer. The radioactive ligand concentration is close to the Kd ([^3^H]-U69593 0.83 nM, [^3^H]-DAMGO 1.20 nM, and [^3^H]-DADLE 2.69 nM). Total binding and nonspecific binding are determined in the absence and presence of 10 μM of appropriate reference compound (Naltrindole DOR, Salvinorin A KOR and DAMGO MOR). In brief, plates are usually incubated at room temperature and in the dark for 90 min. Reactions are stopped by vacuum filtration onto 0.3% polyethyleneimine (PEI) soaked 96-well filter mats using a 96-well Filtermate harvester, followed by three washes with cold PBS buffer. Scintillation cocktail is then melted onto the microwave-dried filters on a hot plate and radioactivity counted in a Microbeta counter. The data (n = 6) were analyzed by nonlinear regression.

### 4.3. BRET Assay 

The BRET recruitment assays were performed with HEK293T cells according to previously reported procedures with minor modifications [26]. Briefly, the cells were co-transfected overnight with Gα_oA_-RLuc, Gβ3, Gγ8-GFP2 and human KOR receptor at a 1:1:1:1 ratio. The next day, cells were seeded (~40,000 cells/well) into poly-L-Lysine coated 96-well white clear bottom cell culture plates in DMEM containing 1% dialyzed FBS. 24 h later, the 96-well plates bottom were covered with white backing (PerkinElmer, Waltham, MA, USA) and the culture medium was removed. Immediately, the cells were washed with 80 µL/well of assay buffer (1× HBSS, 20 mM HEPES, 1 mg/mL BSA, pH 7.4). Then the cells were treated with 80 µL of drugs in assay buffer for 10 min at room temperature, followed by addition of 20 µL/well of RLuc substrate and incubated for another 10 min. Plates were read using a Mithras LB940 reader for the RLuc Luminescence (400 nm) and GFP2 (515 nm) emission and the ratio of GFP2/RLuc (n = 24) was analyzed by non-linear regression using GraphPad Prism 8 (GraphPad Software, San Diego, CA, USA).

### 4.4. Docking

The crystal structure of KOR bound to MP1104 and an active-state-stabilizing nanobody (PDB ID 6B73) [27] was prepared for docking using the Molecular Operating Environment (MOE) structure preparation function to repair any structural defects, adjust partial charges and protonation state, and optimize the hydrogen bond network, hydrogen positions and solvent molecules. For the GL-I-30, U50,488 and MP-II-068 docking, a triangle matcher placement using London dG scoring was performed for 30 poses followed by a refinement using a rigid receptor ad GBVI/WSA dG scoring for 5 poses. The final scorings for GL-I-30 were −8.4 kJ/mol and −8.1 kJ/mol, respectively (Figure 3A,C). The scoring for MP-II-068 was −7.82 kJ/mol (Figure 3D).

## 5. Conclusions

It can be concluded that hydrophobic ester and amide imidazodiazepines with a 2′-fluorophenyl substitution are novel KOR agonists with high selectivity among opioid receptors. A SAR study identified ligands that interacted selectivity with KOR and those binding KOR and BZR enabling diverse therapeutic applications. Elements of structural importance for KOR binding supported a proposed docking model of imidazodiazepines to inform future ligand design. Although complete GPCR signaling is yet to be determined, recruitment of Gα_oA_ to KOR by these ligands reflected the KOR affinity data.

## 6. Patents

The compounds disclosed in the publication are part of the following patents. J.M.C. in: “Selective Agents for Pain Suppression” (US20100317619), “Stereospecific Anxiolytic and Anticonvulsant Agent with Reduced Muscle-Relaxant, Sedative, Hypnotic, and Ataxic Effects” (US20060003995), “Anxiolytic Agents with Reduced Sedative and Ataxic Effects” (US7119196B2) and “GABAergic Agents to Treat Memory Deficits” (US20100130479). J.M.C., M.M.P., K.R.M., and G.L. are inventors of patent: “GABAergic Ligands and Their Uses” (WO2016154031). J.M.C., G.L., and M.M.P. are inventors of “Treatment of Cognitive and Mood Symptoms in Neurodegenerative and Neuropsychiatric Disorders with Alpha-5-containing GABA(A) receptor Agonists” (WO2017161370). G.L., J.M.C., D.C.S. and L.A.A. are inventors of patent “GABA(A) Receptor Modulators and Methods to Control Airway Hyperresponsiveness and Inflammation in Asthma” (WO2018035246A1).

## Figures and Tables

**Table 1:**
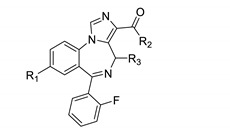
Opioid and benzodiazepine receptor binding of 2′-fluorophenyl substituted imidazodiazepines.

Entry	Compound	R_1_	R_2_	R_3_ CH_3_	KOR % ^1^	MOR % ^1^	DOR % ^1^	BZR % ^1^	KOR (Ki, nM)	BZR (Ki, nM)
1	GL-I-30	HC≡C	*t*-BuO	(S)	95	54	7	96	27	177
2	GL-I-33	HC≡C	*t*-PenO	(S)	94	30	25	96	34	117
3	GL-I-41	HC≡C	*t*-Bu(H)N	(S)	97	40	10	90	39	140
4	GL-I-78	*c*-Pr	EtO	(S)	96	32	41	76	48	352
5	SH-I-048B	Br	EtO	(S)	95	0	0	61	63	96
6	GL-I-32	HC≡C	PrO	(S)	95	9	14	98	64	148
7	GL-I-31	HC≡C	*i-*PrO	(S)	94	32	8	97	65	245
8	GL-I-38	HC≡C	*c*-PrO	(S)	95	18	16	96	68	127
9	SH-I-047	Br	EtO	(R)	82	16	0	84	86	238
10	SH-053-2′F-S-CH_3_	HC≡C	EtO	(S)	93	7	36	92	90	111
11	GL-I-43	HC≡C	Et(H)N	(S)	95	22	3	94	102	44
12	MP-III-023	HC≡C	Me(H)N	(S)	91	0	16	97	119	37
13	MP-III-021	HC≡C	MeO	(S)	93	0	22	88	122	219
14	GL-I-77	HC≡C	EtS	(S)	95	41	4	92	125	124
15	GL-I-55	HC≡C	*c*-Pr(H)N	(S)	93	17	24	95	150	20
16	GL-III-68	*c*-Pr	Et(H)N	(R)	88	0	10	100	150	452
17	GL-III-42	*c*-Pr	EtO	(R)	86	0	13	62	174	726
18	GL-II-74	HC≡C	Et(H)N	(R)	86	10	0	84	194	68
19	GL-III-66	HC≡C	*i-*Pr(H)N	(R)	63	0	0	88	233	271
20	MP-III-058	Br	MeO	(R)	84	0	4	86	237	290
21	SH-053-2′F-R-CH_3_	HC≡C	EtO	(R)	89	28	32	85	240	379
22	GL-II-75	HC≡C	*c*-Pr(H)N	(R)	81	0	3	85	278	93
23	GL-II-76	HC≡C	Pyrrolidine	(R)	80	0	0	43	371	- ^2^
24	MP-III-022	HC≡C	Me(H)N	(R)	80	3	22	95	381	83
25	GL-I-36	HC≡C	F_3_CCH_2_O	(S)	85	8	0	77	411	418
26	GL-III-69	Br	Me_2_N	(R)	75	0	10	100	511	446
27	MP-II-075	HC≡C	BzO	H	84	0	16	98	547	21
28	MP-III-004	HC≡C	MeO	(R)	76	0	24	78	599	445
29	GL-I-54	HC≡C	Me_2_N	(S)	78	18	11	94	788	90
30	GL-III-70	*c*-Pr	Me_2_N	(R)	68	0	50	100	800	3395
31	GL-II-73	HC≡C	Me_2_N	(R)	58	6	0	75	1189	506
32	MP-III-019.B	HC≡C	H_2_N	(R)	62	2	0	94	1534	54
33	MP-III-018.B	HC≡C	H_2_N	(S)	51	2	10	97	2782	17
34	GL-III-54	Cl	HO	(R)	22	0	0	100	- ^2^	42
35	SH-053-2′F-S-CH_3_-Acid	HC≡C	HO	(S)	20	0	18	93	- ^2^	29
36	SH-053-2′F-R-CH_3_-Acid	HC≡C	HO	(R)	16	0	0	93	- ^2^	37
37	GL-II-93	Br	HO	(R)	0	0	0	73	- ^2^	86

^1^ Percent inhibition at 10,000 nM; ^2^ dose response was carried out only for compounds with an inhibition of >50%.

**Table 2:**
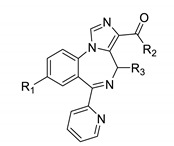
Opioid and benzodiazepine receptor binding of 2′-pyridine substituted imidazodiazepines.

Entry	Name	R_1_	R_2_	R_3_ CH_3_	KOR ^1^ %	MOR ^1^ %	DOR ^1^ %	BZR ^1^ %	KOR (Ki, nM)	BZR (Ki, nM)
1	MP-II-068	HC≡C	iPrO	H	85	0	34	97	550	42
2	GL-II-06	Br	EtO	(R)	79	11	9	81	401	556
3	Hz-166	HC≡C	EtO	H	60	6	49	n.d.	3821	n.d.
4	GL-II-19	HC≡C	EtO	(R)	44	17	2	71	- ^2^	1143
5	MP-III-024	HC≡C	MeO	H	43	0	24	81	- ^2^	277
6	GL-II-32	HC≡C	MeO	(R)	35	32	0	63	- ^2^	1427
7	GL-II-31	HC≡C	MeHN	(R)	23	17	3	72	- ^2^	1697
8	GL-II-51	Br	HO	(R)	11	0	0	84	- ^2^	181
9	SR-II-54	HC≡C	HO	H	9	8	1	80	- ^2^	69
10	GL-II-30	HC≡C	HO	(R)	0	20	4	65	- ^2^	431

^1^ Percent inhibition at 10,000 nM, ^2^ dose response was carried out only for compounds with an inhibition of >50%, n.d. = not determined.

**Table 3:**
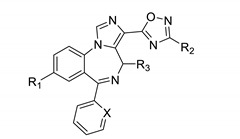
Opioid and benzodiazepine receptor binding of imidazodiazepine oxadiazoles.

Entry	Name	R_1_	R_2_	R_3_	X	KOR ^1^ %	MOR ^1^ %	DOR ^1^ %	BZR ^1^ %	KOR (Ki, nM)	BZR (Ki, nM)
1	GL-I-81	HC≡C	iPr	(S)	C-F	93	64	25	83	127	839
2	MP-IV-010	HC≡C	iPr	(R)	C-F	94	33	5	64	145	814
3	GL-I-66	HC≡C	Et	(S)	C-F	92	28	2	80	212	654
4	GL-I-65	HC≡C	Me	(S)	C-F	88	2	7	79	222	1147
5	GL-IV-03	*c*-Pr	Et	(R)	C-F	91	25	67	65	232	n.d.
6	GL-III-60	Br	Me	(R)	C-F	87	26	18	100	232	784
7	MP-IV-004	HC≡C	Me	(R)	C-F	86	7	10	63	444	1079
8	GL-II-54	HC≡C	Et	(R)	N	80	0	0	66	504	2037
9	GL-III-63	*c*-Pr	Me	(R)	C-F	78	8	31	28	678	- ^2^
10	GL-III-64	*c*-Pr	Me	(R)	N	60	7	14	92	2048	1490
11	MP-III-085	HC≡C	Me	H	N	40	0	0	82	- ^2^	357
12	MP-III-080	HC≡C	Et	H	N	47	0	0	88	- ^2^	303

^1^ Percent inhibition at 10,000 nM, ^2^ dose response was carried out only for compounds with an inhibition of >50%, n.d. = not determined.

**Table 4:**
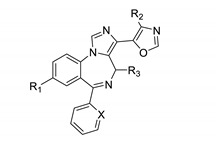
Opioid and benzodiazepine receptor binding of imidazodiazepine oxazoles.

Entry	Name	R_1_	R_2_	R_3_	X	KOR ^1^ %	MOR ^1^ %	DOR ^1^ %	BZR ^1^ %	KOR (Ki, nM)	BZR (Ki, nM)
1	SH-I-85	Br	H	H	C-F	91	16	33	99	162	11
2	GL-III-76	Br	H	(S)	C-F	83	3	0	100	221	120
3	GL-III-36	Br	H	(R)	C-F	88	0	12	86	246	319
4	KRM-II-18B	HC≡C	H	H	C-F	89	11	20	97	361	38
5	KRM-III-59	Br	Me	H	C-F	73	0	11	96	408	20
6	GL-III-73	HC≡C	H	(R)	C-F	74	12	4	65	449	602
7	GL-III-78	HC≡C	H	(S)	C-F	74	10	9	101	451	261
8	KRM-II-73	Br	H	H	C-H	83	11	32	97	736	45
9	KRM-III-66	Br	Me	H	C-H	67	12	11	86	756	49
10	KRM-III-65	HC≡C	Me	H	C-F	64	0	16	96	879	45
11	KRM-II-82	HC≡C	H	H	C-H	78	5	20	95	1203	96
12	KRM-II-97	Br	H	H	N	62	18	8	93	1959	140
13	KRM-III-67	HC≡C	H	H	N	48	30	3	71	- ^2^	144
14	KRM-II-81	Br	Me	H	N	46	7	15	86	- ^2^	294
15	KRM-III-79	HC≡C	Me	H	N	9	32	1	81	- ^2^	308

^1^ Percent inhibition at 10,000 nM, ^2^ dose response was carried out only for compounds with an inhibition of >50%.
